# The ARTS of p53-dependent mitochondrial apoptosis

**DOI:** 10.1093/jmcb/mjac074

**Published:** 2022-12-24

**Authors:** Qian Hao, Jiaxiang Chen, Hua Lu, Xiang Zhou

**Affiliations:** Fudan University Shanghai Cancer Center and Institutes of Biomedical Sciences, Fudan University, Shanghai 200032, China; Department of Oncology, Shanghai Medical College, Fudan University, Shanghai 200032, China; Department of Physiology, Medical College of Nanchang University, Nanchang 330006, China; Jiangxi Provincial Key Laboratory of Reproductive Physiology and Pathology, Nanchang University, Nanchang 330006, China; Department of Biochemistry & Molecular Biology and Tulane Cancer Center, Tulane University School of Medicine, New Orleans, LA 70112, USA; Fudan University Shanghai Cancer Center and Institutes of Biomedical Sciences, Fudan University, Shanghai 200032, China; Department of Oncology, Shanghai Medical College, Fudan University, Shanghai 200032, China; Key Laboratory of Breast Cancer in Shanghai, Fudan University Shanghai Cancer Center, Fudan University, Shanghai 200032, China; Shanghai Key Laboratory of Medical Epigenetics, International Co-laboratory of Medical Epigenetics and Metabolism (Ministry of Science and Technology), Institutes of Biomedical Sciences, Fudan University, Shanghai 200032, China

**Keywords:** p53, ARTS, SEPT4, BCL-2 family, apoptosis, cancer therapy

## Abstract

The tumor-suppressive activity of p53 is largely attributed to its ability to induce cell death, including apoptosis, through transcription-dependent and transcription-independent mechanisms. On the one hand, nuclear p53 transcriptionally activates the expression of a myriad of pro-apoptotic BCL-2 family genes, such as *NOXA, PUMA, BID, BAD, BIK, BAX*, etc., whereas it inactivates the expression of anti-apoptotic *BCL-2, BCL-X_L_*, and *MCL1*, leading to mitochondrial apoptosis. On the other hand, cytoplasmic p53 also promotes mitochondrial apoptosis by directly associating with multiple BCL-2 family proteins in the mitochondria. Apoptosis-related protein in TGF-β signaling pathway (ARTS), a mitochondria-localized pro-apoptotic protein encoded by an alternative spliced variant of the *SEPT4* gene, triggers apoptosis by facilitating proteasomal degradation of BCL-2 and XIAP upon pro-apoptotic stimuli. We recently identified *SEPT4/ARTS* as a new p53 target gene in response to genotoxic stress. ARTS in turn binds to p53, drives its mitochondrial localization, and enhances the interaction between p53 and BCL-X_L_, thereby promoting mitochondrial apoptosis. This review will illustrate the mechanisms of p53-induced mitochondrial apoptosis, offer some recently discovered new insights into the functions of ARTS in regulating mitochondrial cell death, and discuss the clinical significance of ARTS in cancer and non-cancer diseases.

## Introduction

The fine balance between cell survival and death is vital for organism growth and development. However, disruption of this balance leads to various diseases, including cancer. Programmed cell death was first observed in the development of toads in the 1840s (Cotter, 2009). One of the major forms of programmed cell death is apoptosis, which can be initiated through either the extrinsic or the intrinsic pathway. The protease activity of caspases is crucial to the morphological and biochemical changes of apoptotic cells (Marino et al., 2014; Ichim and Tait, 2016). In the extrinsic apoptotic pathway, the death receptors upon binding by their cognate ligands can activate the initiator caspases, caspase-8 and caspase-10, which in turn mediate the cleavage of the effectors, caspase-3, caspase-6, and caspase-7, consequently leading to apoptosis (Ichim and Tait, 2016). The intrinsic apoptotic pathway, also known as mitochondrial apoptosis, involves a complex interplay between the pro-apoptotic and anti-apoptotic BCL-2 family proteins (Singh et al., 2019). Upon various stress signals, the pro-apoptotic BCL-2 homology domain 3 (BH3)-only proteins, including BCL-2-associated agonist of cell death (BAD), BH3-interacting domain death agonist (BID), BCL-2-interacting killer (BIK), BCL-2-interacting mediator of cell death (BIM), BCL-2-modifying factor (BMF), activator of apoptosis harakiri (HRK), phorbol-12-myristate-13-acetate-induced protein 1 (PMAIP1, also known as NOXA), and p53-upregulated modulator of apoptosis (PUMA), can be triggered to interact with and activate the pore-forming proteins, BCL-2-associated X protein (BAX) and BCL-2 antagonist/killer 1 (BAK, also known as BAK1), at the outer mitochondrial membrane, leading to mitochondrial outer membrane permeabilization (MOMP) and the release of cytochrome c, second mitochondria-derived activator of caspases (SMAC), and serine protease HTRA2/OMI. Cytochrome c associates with apoptotic peptidase activating factor 1 (APAF1) in the cytoplasm to form the apoptosome and facilitate caspase-9 activation, while SMAC and HTRA2/OMI suppress the anti-apoptotic X-linked inhibitor of apoptosis protein (XIAP). These signals are committed to mediating the cleavage of the effector caspases and triggering apoptosis (Singh et al., 2019). To circumvent apoptosis and sustain their own survival and propagation, cancer cells employ the multi-BH domain-containing anti-apoptotic BCL-2 family proteins, such as BCL-2, BCL-X_L_ (the longer isoform of BCL2L1), and MCL1, to repress the pro-apoptotic BCL-2 family proteins by directly associating with the latter (Green, 2022). This complicated apoptotic process is finely tuned by various regulators in cancer cells.

**Figure 1:**
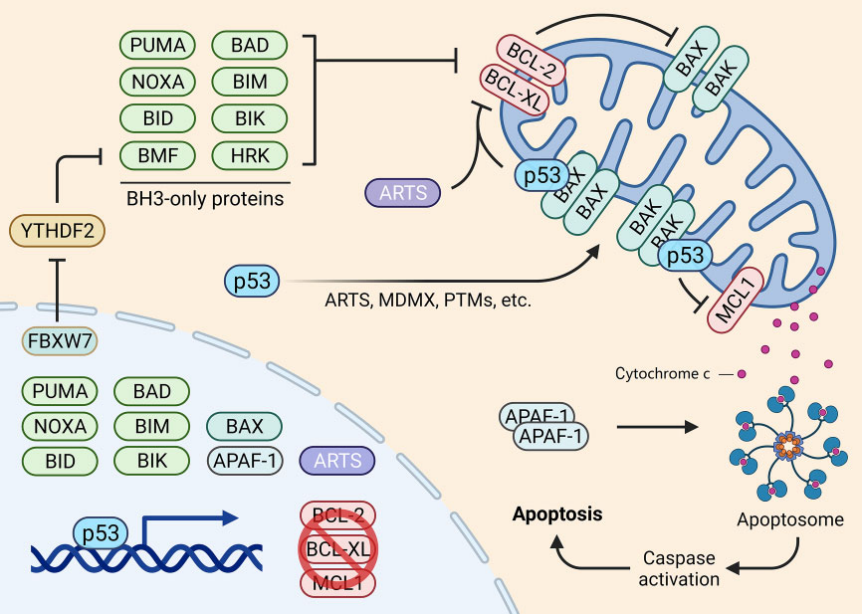
p53-dependent mitochondrial apoptotic signaling network. Nuclear p53 transcriptionally activates the expression of PUMA, NOXA, BID, BAD, BIM, BIK, BAX, and APAF1, but represses the expression of BCL-2, BCL-X_L_, and MCL1. In addition, p53 may induce BMF expression through the FBXW7–YTHDF2 cascade. With the aid of ARTS, MDMX, and PTMs, cytoplasmic p53 can translocate to the mitochondria to interact with BAX, BAK, BCL-2, BCL-X_L_, and MCL1, consequently inducing apoptosis.

One of the important regulators is the tumor suppressor p53. p53 is regarded as the ‘guardian of the genome’ because of its important role in preventing tumorigenesis and inhibiting cancer progression (Levine, 2020). In response to various stresses, p53 is activated through different post-translational modifications (PTMs) (Liu et al., 2019; Wen and Wang, 2022) to act as either a transcription factor in the nucleus (Riley et al., 2008) or an apoptosis inducer in the cytoplasm (Green and Kroemer, 2009). On the one hand, p53 suppresses cancer development by transcriptionally regulating the expression of myriad genes (Riley et al., 2008). These downstream target genes are responsible for different biological processes, such as cell cycle arrest, DNA repair, and apoptosis (Levine, 2019, 2020). On the other hand, the cytoplasmic p53 protein induces apoptosis by directly interacting with multiple BCL-2 family proteins (Green and Kroemer, 2009). However, p53-dependent apoptosis is causative for developmental abnormalities when p53 is inappropriately activated during embryonic and postnatal development (Bowen and Attardi, 2019). For instance, germline mutations or haploinsufficiency of ribosome biogenesis-associated genes cause ribosomopathies that compose a group of developmental disorders characterized by reduced viability and population of erythroid precursors, neural crest cells, or other tissue-specific cell types (Zhou et al., 2015; Calo et al., 2018). Consistently, multiple mouse strains with increased p53 activity due to conventional or conditional inactivation of *Mdm2* and/or *Mdmx*, which encode two master inhibitors of p53, display diverse developmental defects (Bowen and Attardi, 2019). The pro-apoptotic function of p53 was first described by the Oren group in 1991 (Yonish-Rouach et al., 1991), which was validated later in mouse embryonic fibroblasts (MEFs) (Lowe et al., 1993) and cancer cells (Aubrey et al., 2018) and will be further discussed below (Figure 1). Recently, we have identified a new target gene of p53, *SEPT4*/*ARTS*, which encodes a pro-apoptotic protein that directly binds to p53 and enhances the interaction between p53 and BCL-X_L_ at the mitochondria, resulting in augmented apoptosis (Hao et al., 2021a). In this review, we illustrate the mechanisms underlying p53 regulation of apoptosis, provide an updated overview of the function of apoptosis-related protein in TGF-β signaling pathway (ARTS) as a critical component of the p53-mediated apoptotic pathway, and discuss the clinical relevance of ARTS in cancer and non-cancer diseases.

## p53-dependent transcriptional regulation of the BCL-2 family genes

p53’s function is mainly executed through its transcriptional activity, because it can induce or repress gene expression by binding to the responsive elements (REs) on gene promoters. A typical RE sequence is degenerate and composed of two ‘half-sites’, 5′-RRRCWWGYYY-3′, which are separated by a spacer of 0–21 base pairs (Riley et al., 2008). *NOXA* was the first BH3-only gene identified as a direct transcriptional target of p53 (Oda et al., 2000). The expression of *Noxa* was found to be upregulated by X-ray irradiation in MEFs and thymocytes but not in p53-deficient (p53^−/−^) murine cells. After induction, Noxa translocates to the mitochondria and interacts with the anti-apoptotic BCL-2 members, leading to caspase-9 activation and apoptosis. Depletion of *Noxa* renders significant resistance of MEFs to DNA damage-induced and p53-mediated apoptosis (Shibue et al., 2003). *PUMA*, also known as *BBC3*, was later found to be another BH3-only gene critical for p53-induced apoptosis by three independent groups (Han et al., 2001; Nakano and Vousden, 2001; Yu et al., 2001). PUMA binds to and inhibits BCL-2 and BCL-X_L_ at the mitochondria, consequently leading to cytochrome c release and APAF1-dependent apoptosis (Han et al., 2001; Nakano and Vousden, 2001; Yu et al., 2001). Genetic studies using *Noxa* or *Puma* knockout mice revealed that Puma acts as a more powerful mediator of p53-dependent apoptosis, as only loss of Puma can protect lymphocytes and hematopoietic cells from apoptosis induced by DNA damage stress (Jeffers et al., 2003; Villunger et al., 2003). Another study using combined knockout mouse models demonstrated that, although both Noxa and Puma are required for p53-induced apoptosis in murine fibroblasts and thymocytes, Puma has a predominant pro-apoptotic function in many other cell types (Michalak et al., 2008). Consistently, p53-dependent PUMA induction leads to chemotherapy-induced intestinal injury, while inhibition of PUMA protects intestinal stem cells against apoptosis (Leibowitz et al., 2018). The critical role of PUMA in p53-mediated apoptosis may be also reflected by the fact that the pseudo-caspase FLIP(L) suppresses apoptosis by selectively inhibiting p53 induction of *PUMA*, but not other pro-apoptotic target genes, such as *NOXA* and *BAX* (Lees et al., 2020). An exception is that loss of Noxa can inhibit UV radiation-induced apoptosis in murine fibroblasts and keratinocytes more dramatically than loss of Puma (Naik et al., 2007), suggesting that NOXA and PUMA may coordinate with each other to regulate apoptosis in the context of different cell types and cellular stresses (El-Saafin et al., 2022). These two BH3-only proteins are both critically important to p53-induced apoptosis, because their combined depletion can prevent murine cells from apoptosis as effectively as knockout of p53 (Michalak et al., 2008).

Besides these two pro-apoptotic genes, p53 can transcriptionally induce more BH3-only genes, including *BID* (Sax et al., 2002), *BIK* (Mathai et al., 2002), and *BAD* (Jiang et al., 2006). *BIM* was initially considered an indirect p53 target gene, as its expression could only be induced after 6 h treatment of etoposide, which was much later than the induction of *PUMA* and *NOXA* (Happo et al., 2010). However, a later study suggested that p53 may directly regulate *BIM* expression via a potential p53-RE on its promoter region through a genome-wide analysis (Kenzelmann Broz et al., 2013). *BMF* could be upregulated by acetylated p53, as IFN-γ-mediated HDAC1 deacetylation of p53 leads to reduced expression of *BMF* (Contreras et al., 2013). We recently found that the E3 ubiquitin ligase FBXW7 promotes proteolytic degradation of the m^6^A-binding protein YTHDF2, leading to stabilization of m^6^A-modified *BMF* mRNA (Xu et al., 2021). Given that p53 transcriptionally activates *FBXW7* expression (Mao et al., 2004; Perez-Losada et al., 2005), our study implies that *BMF* may be an indirect target gene of p53. In addition, p53 represses the transcription of the anti-apoptotic members, *BCL-2, BCL-X_L_*, and *MCL1*, through indirect mechanisms (Miyashita et al., 1994; Sugars et al., 2001; Pietrzak and Puzianowska-Kuznicka, 2008; Aubrey et al., 2022). Collectively, p53 can promote mitochondrial apoptosis by either activating the expression of the pro-apoptotic BCL-2 family genes or inhibiting the expression of the anti-apoptotic genes.

Inhibition of the anti-apoptotic BCL-2 proteins leads to the activation of the pore-forming proteins, BAX and BAK, which is a prerequisite for MOMP and the release of cytochrome c (Oltvai et al., 1993; Chittenden et al., 1995; Farrow et al., 1995; Kiefer et al., 1995). BCL-2 and BCL-X_L_ inactivate the pore-forming proteins by forming heterodimers with the latter (Yin et al., 1994; Farrow et al., 1995). When cells are under apoptotic stimulation, however, the BH3-only proteins bind to BAX and BAK to facilitate their homo-oligomerization, leading to the pore formation at the mitochondrial outer membrane and consequent cytochrome c release (Czabotar et al., 2013). Interestingly, a putative p53-RE was found on the *BAX* gene promoter, which was responsible for *BAX* transcription upon genotoxic stress in a p53-dependent fashion (Miyashita et al., 1994; Selvakumaran et al., 1994; Zhan et al., 1994; Miyashita and Reed, 1995). Recently, we unveiled an additional mechanism accounting for p53-induced *BAX* transcription (Liao et al., 2016). The transcription elongation factor TFIIS.h, which is encoded by the *TCEA3* gene that is transcriptionally induced by p53, specifically associates with the genomic DNA and the transcripts of *BAX*, thereby enhancing its transcription (Liao et al., 2016). Thus, p53 can induce *BAX* mRNA expression by activating its transcription initiation and enhancing its transcription elongation via distinct mechanisms. Finally, p53 transcriptionally activates the APAF1-encoding gene, whose protein product can serve as a scaffold for apoptosome assembly and caspase activation (Soengas et al., 1999; Fortin et al., 2001; Kannan et al., 2001; Moroni et al., 2001; Robles et al., 2001). Taken together, these studies demonstrate that p53 can regulate the expression of a wide range of genes involved in the multistep mitochondrial apoptosis, including activation of pro-apoptotic and inhibition of anti-apoptotic BCL-2 proteins, MOMP, cytochrome c release, apoptosome assembly, and caspase activation (Figure 1).

## Regulation of the BCL-2 family proteins by cytosolic p53

The transcription-independent pro-apoptotic activity of p53 was first described in the middle of the 1990s, as evidenced by the fact that several p53 mutants with deficiencies in transcription activity could still trigger apoptosis (Caelles et al., 1994; Haupt et al., 1995). Stress-activated p53 was found to translocate to the mitochondria, thereby eliciting cytochrome c release and caspase activation in both primary and cancer cells (Marchenko et al., 2000; Mihara et al., 2003). Mechanistically, mitochondrial p53, like many other BH3-only proteins, can form complexes with and, as such, inhibit the apoptotic antagonists, BCL-2 and BCL-X_L_ (Mihara et al., 2003; Wei et al., 2021). This action may be facilitated by the MDM2 homolog, MDMX, as it promotes the translocation of cytoplasmic p53 to the mitochondria and enhances p53 interaction with BCL-2 (Mancini et al., 2009). Interestingly, the polymorphic variants of p53 were shown to have distinct accessibility to the mitochondria—the arginine-72 variant of p53 exhibits greater potential to trigger cytochrome c release than the proline-72 variant (Dumont et al., 2003; Almeida et al., 2021). Conversely, overexpression of the anti-apoptotic BCL-2 members can inhibit p53-induced apoptosis as well (Strasser et al., 1994; Chipuk et al., 2005). BCL-X_L_ was found to bind to the DNA-binding domain (DBD) of p53 (Follis et al., 2014) and block cytoplasmic p53 from triggering apoptosis upon inhibition of EGFR-driven glucose metabolism, which leads to an effective combination therapy by targeting EGFR and pharmacologically stabilizing p53 (Mai et al., 2017). In addition, p53 directly binds to and activates the pore-forming protein, BAX, and depletion of BAX completely abrogates cytoplasmic p53-induced apoptosis in cancer cells and MEFs (Chipuk et al., 2003, 2004). p53 was also found to promote the translocation of BAX from the cytoplasm to the mitochondria through their direct interaction (Dubrez et al., 2001; Schuler et al., 2003). A later study showed that *cis–trans* isomerization of proline 47 within the p53 protein catalyzed by the prolyl isomerase PIN1 is required for BAX activation (Follis et al., 2015). Another pore-forming protein, BAK, is also critical for the pro-apoptotic function of cytoplasmic p53. p53 interacts with BAK at the mitochondria to facilitate homo-oligomerization of the latter through perturbation of the MCL1/BAK interaction, resulting in MOMP and cytochrome c release (Leu et al., 2004), while TRAF6-mediated p53 ubiquitination prevents its mitochondrial translocation and the interaction between p53 and MCL1/BAK (Zhang et al., 2016). Additionally, p53 can mediate the proteasomal degradation of MCL1, though the underlying mechanism is not well understood. Activation of p53 by the MDM2 inhibitor, RG7388, modulates phosphorylation of MCL1 and promotes its proteasomal degradation, thus overcoming apoptosis resistance of acute myeloid leukemia (Pan et al., 2017). Recently, p53 was also found to promote MCL1 degradation, resulting in the release of BAK and induction of apoptosis, in MYC-driven B-cell lymphomas (Domostegui et al., 2021). An interesting observation is that cancer-associated mutations in the DBD of p53 impair the pro-apoptotic activity of cytoplasmic p53 (Zhang et al., 2020), as they disrupt the interactions of BCL-X_L_ and MCL1 with p53’s DBD (Mihara et al., 2003; Leu et al., 2004; Pietsch et al., 2008). Together, cytoplasmic p53 participates in mitochondrial apoptosis by physically interacting with and regulating BCL-2 family proteins (Figure 1).

## The role of ARTS in promoting mitochondrial apoptosis

**Figure 2:**
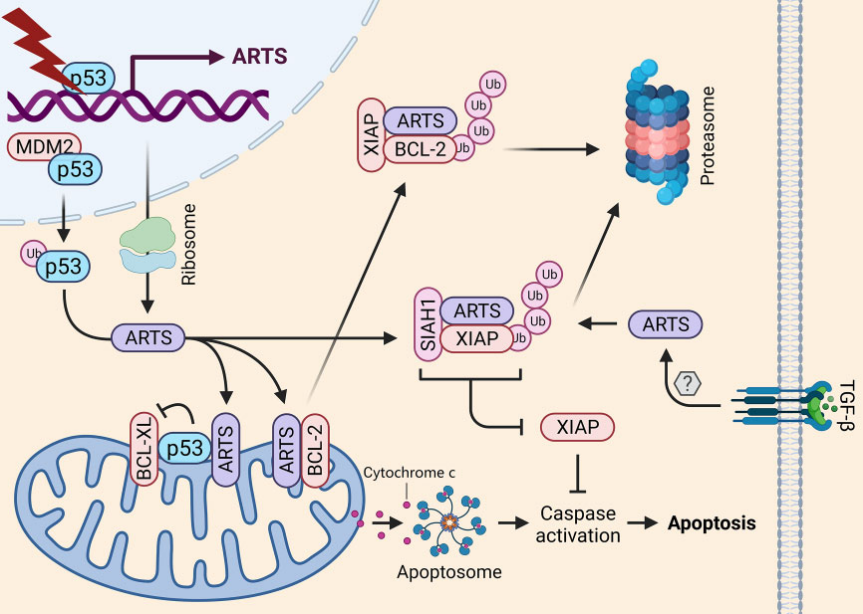
The role of ARTS in promoting mitochondrial apoptosis. ARTS is encoded by a p53-target gene whose expression is responsive to diverse apoptotic stimuli, such as DNA damage stress and TGF-β activation. There are three mechanisms for ARTS-induced mitochondrial apoptosis: (i) ARTS facilitates the mitochondrial localization of p53 and enhances the p53–BCL-X_L_ interaction at the mitochondria; (ii) ARTS suppresses XIAP and promotes its proteasomal degradation by recruiting SIAH1 as a ternary complex; and (iii) ARTS bridges BCL-2 and XIAP by directly binding to them, allowing for XIAP-mediated proteasomal degradation of BCL-2.

ARTS protein, encoded by an alternative spliced variant of the *SEPT4* gene, is located at the outer membrane of the mitochondria (Figure 2; Larisch et al., 2000; Mandel-Gutfreund et al., 2011). ARTS was originally identified as a TGF-β-responsive protein by retroviral insertional mutagenesis screening, because overexpression of ARTS enhanced, whereas depletion of ARTS inhibited, TGF-β-induced caspase activation and apoptosis (Larisch et al., 2000). Later studies showed that ARTS can also be activated by a variety of pro-apoptotic stimuli, including staurosporine, arabinoside, 5-azacytidine, etoposide, and UV irradiation (Elhasid et al., 2004; Gottfried et al., 2004; Edison et al., 2012b). One of the mechanisms underlying the pro-apoptotic activity of ARTS is through inhibition of XIAP (Gottfried et al., 2004). Upon pro-apoptotic stimuli, a portion of ARTS molecules are released from the mitochondria to the cytoplasm, where they bind to the BIR1 and BIR3 domains of XIAP (Gottfried et al., 2004; Bornstein et al., 2011). Also, ARTS binds to and recruits the E3 ubiquitin ligase SIAH1 to promote ubiquitination and degradation of XIAP (Garrison et al., 2011). The translocation of ARTS to the cytoplasm precedes the release of cytochrome c and SMAC, which is therefore considered a critical step for apoptosis initiation (Edison et al., 2012b). Recently, ARTS was found to directly associate with the BH3 domain of BCL-2 and promote XIAP-induced BCL-2 proteasomal degradation (Edison et al., 2017). The physiological function of ARTS has also been extensively studied in multiple genetic mouse models, demonstrating that this apoptosis inducer plays essential roles in spermatogenesis, development of hematopoietic and intestinal stem cells, and skin regeneration (Kissel et al., 2005; Garcia-Fernandez et al., 2010; Fuchs et al., 2013; Koren et al., 2018). ARTS expression is controlled by surveillance mechanisms that maintain a low level of ARTS to support cell survival, while the ubiquitin-mediated degradation of ARTS is repressed when cells are under pro-apoptotic stimuli or DNA damage stress (Lotan et al., 2005). The apoptosis inhibitor, XIAP, which is repressed and degraded by ARTS (Gottfried et al., 2004; Bornstein et al., 2011; Garrison et al., 2011), was identified as an E3 ubiquitin ligase targeting ARTS for proteasomal degradation, thus forming a negative feedback circuit (Bornstein et al., 2012). In addition, the E3 ubiquitin ligase, Parkin, which is encoded by the Parkinson's disease-associated gene, *PRKN*, was found to promote ARTS ubiquitination and degradation in neuronal cells and rat brains (Kemeny et al., 2012). This finding may partially explain why mutations of *PRKN* result in neuronal cell death and neurodegeneration.

Recently, we identified *SEPT4/ARTS* as a p53-responsive gene that plays a critical role in p53-dependent mitochondrial apoptosis (Figure 2; Hao et al., 2021a). To elucidate the tumor-suppressive functions of p53, we performed a microarray analysis to screen the significant differentially expressed genes in colon cancer HCT116 cells treated with or without the p53-inducing agent, Inauhzin (Liao et al., 2012; Zhang et al., 2012). This screening led to the identification of several important transcriptional target genes of p53, such as *NGFR* (Zhou et al., 2016), *PHLDB3* (Chao et al., 2016), *TCEA3* (Liao et al., 2016), and *SEPT4*/*ARTS* (Hao et al., 2021a). To support this, treatment of cancer cells with various p53-inducing agents, including cisplatin, doxorubicin, 5-fluorouracil, and Nutlin-3, or overexpression of p53 in cancer cells, resulted in the increase of both mRNA and protein levels of ARTS. Additionally, γ-irradiation dramatically boosted *Sept4*/*Arts* expression in thymuses and spleens of p53^+/+^, but not p53^−/−^, mice. Importantly, a p53-RE at −2279 bp upstream of the transcription initiation site was validated through the luciferase reporter assay and the chromatin IP assay, demonstrating *SEPT4*/*ARTS* as a *bona fide* p53 target gene. Intriguingly, we accidentally found that mutant p53 might bind to a peptide (KLQDQALKE) encoded by the *SEPT4* gene in ovarian cancer in another study (Chen et al., 2019). This prompted us to test whether wild-type p53 can also bind to ARTS, because both mutant and wild-type p53 share many common binding partners (Hao et al., 2020). Indeed, we further confirmed that p53 interacts with ARTS in cancer cells and, more importantly, at the mitochondria. Unlike NGFR (Zhou et al., 2016) and PHLDB3 (Chao et al., 2016), ARTS did not regulate p53 protein stability, as enforced expression of ARTS failed to affect the expression of exogenous and endogenous p53 in H1299 and HCT116 cells, respectively. Instead, ARTS facilitated p53 translocation to the mitochondria and increased the interaction of p53 with BCL-X_L_, consequently leading to inhibition of BCL-X_L_ and augmented apoptosis. Remarkably, our study also revealed ARTS as a biomarker for tumor chemosensitivity, dependent on p53, because overexpression of ARTS enhanced, whereas depletion of ARTS impaired, chemotherapy-induced apoptosis in wild-type p53-harboring cancer cells (Hao et al., 2021a). Together, our findings unveil ARTS as a new p53 target and partner in the p53-mediated mitochondrial apoptotic pathway.

## Relevance of ARTS in cancer and non-cancer diseases

Since ARTS is an apoptosis inducer by antagonizing the anti-apoptotic proteins, such as XIAP, BCL-2, and BCL-X_L_, as described above (Figure 2), it might act as a tumor suppressor. Supporting this speculation are the following lines of evidence. First, ARTS was found to be underexpressed in lymphoblasts of >70% of childhood acute lymphoblastic leukemia (ALL) patients (Elhasid et al., 2004). This cancer-associated deficiency was specific to the ARTS isoform, as the expression of H5, another variant encoded by *SEPT4*, was not affected, suggesting that an RNA splicing mechanism might be dysregulated in these patients. After treatment with chemotherapy, the levels of ARTS in lymphocytes were increased by 2–30 folds, which was correlated with disease remission. Also, leukemic cells lacking ARTS expression are resistant to chemotherapy-induced apoptosis. These findings support the idea that ARTS acts as both a tumor suppressor and a prognostic biomarker, at least, for ALL. The tumor-suppressive role of ARTS was further validated by a study using a *Sept4*-deficient mouse model (Garcia-Fernandez et al., 2010). Deletion of *Sept4* led to an increased number of hematopoietic stem cells or tumor-initiating cells, upregulation of XIAP, resistance to apoptosis, and, consequently, accelerated development of leukemias and lymphomas. The findings suggest that ARTS mimetics or activators might serve as potential therapeutic agents for cancers with high levels of XIAP, BCL-2, or BCL-X_L_ (Shahar and Larisch, 2020). The C-terminus of ARTS contains a unique 27-residue peptide, which is distinct from other known proteins (Edison et al., 2012a). Subdivision of this peptide revealed that a 9-residue peptide is sufficient for binding to the BIR3 domain of XIAP and inducing caspase activation and apoptosis. Recently, a candidate compound that mimics ARTS to specifically bind to XIAP was identified through a structure-based computational screen (Mamriev et al., 2020). The compound can induce apoptosis by promoting the degradation of both XIAP and BCL-2. In addition, loss of ARTS expression in leukemia is partially due to epigenetic silencing by DNA methylation (Elhasid et al., 2004). The methylation inhibitor, 5-azacytidine, was shown to elevate the expression of ARTS in both leukemic cell lines and ALL patients. Furthermore, genotoxic agents or p53-inducing agents can induce the expression of ARTS, consistent with our findings as described above (Hao et al., 2021a). It is thus speculated that the combination of methylation inhibitors, which remove DNA methylation from the *SEPT4*/*ARTS* promoter, with genotoxic agents, which induce p53 activation (Hao et al., 2021b), could be a more effective approach for the treatment of cancers, such as leukemia, which sustain wild-type p53 and lower levels of ARTS, by inducing ARTS expression and ARTS-dependent apoptosis. Hence, further exploring this translational potential in the near future would be tremendously conducive to the development of a new strategy for anti-cancer therapies.

In addition, ARTS induction may contribute to developmental defects caused by aberrant p53 activation, as depletion of *Sept4*/*Arts* promotes the development and renewal of several types of stem cells, including germinal, hematopoietic, and cutaneous stem cells, by inhibiting apoptosis (Kissel et al., 2005; Garcia-Fernandez et al., 2010; Fuchs et al., 2013). Thus, the development of ARTS antagonists could be helpful for treating subsets of ribosomopathies that are caused by tissue-specific activation of p53 (Zhou et al., 2015; Calo et al., 2018). Another possible clinical application of ARTS inhibition might prevent intestinal injury triggered by cancer chemotherapies. Most chemotherapeutic agents can cause intestinal dysfunction or enterotoxicity that is associated with intestinal crypt apoptosis (Leibowitz et al., 2018). Since knockout of *Sept4*/*Arts* promotes the renewal of Lgr5^+^ intestinal stem cells and thus the regeneration of crypts (Koren et al., 2018), targeting intestinal ARTS could be a promising strategy for alleviating normal intestinal damage without affecting the pro-apoptotic activity of p53 in cancer cells.

## Notes

**Author contributions:** Q.H., J.C., and X.Z. drafted the manuscript; H.L. and X.Z. revised the manuscript.
